# Land system changes of terrestrial tipping elements on Earth under global climate pledges: 2000–2100

**DOI:** 10.1038/s41597-025-04444-8

**Published:** 2025-01-27

**Authors:** Jiaying Lv, Yifan Gao, Changqing Song, Li Chen, Sijing Ye, Peichao Gao

**Affiliations:** State Key Laboratory of Earth Surface Processes and Resource Ecology, Beijing Normal University, Beijing, 100875 China

**Keywords:** Environmental impact, Projection and prediction

## Abstract

Tipping elements on Earth are components that undergo rapid and irreversible changes when climate change reaches a tipping point. They are highly sensitive to climate variations and serve as early warning signs of global change. Human activities, including global climate pledges, significantly influence the climate and the state of tipping elements. Land changes serve as the external and intuitive response of tipping elements to climate change, making it essential to identify shifts in the land system. We produced a 1-km land system dataset for terrestrial tipping elements on Earth for the years 2000, 2010, 2020, and 2100 under global climate pledges by integrating the GCAM with a modified version of CLUMondo. Our dataset includes 30 thematic categories, combining three density types and ten land cover types. The dataset illustrates potential land system changes under global climate pledges, contrasting with common SSP and RCP scenarios. Our simulations demonstrate high accuracy, offering valuable insights into tipping elements and the assessment of the impacts of global climate pledges on Earth.

## Background & Summary

Earth is a highly complex adaptive system composed of diverse structures and components, including subsystems such as the geosphere, atmosphere, hydrosphere, cryosphere, biosphere, and socioeconomic systems. These subsystems interact through the exchange of energy, information, and matter^1^ and are characterized by nonlinear feedback, multiple interactions, and intricate structural relationships, which allow Earth to be regarded as a self-regulating system^2^. In recent years, there has been a growing focus on Earth system science^3^, which investigates the dynamic processes, interactions, and statistical characterizations within these systems^1,4–6^. This field faces the challenge of integrating biophysical processes with human dynamics^3^, making in-depth studies of Earth system science crucial for the sustainable development of human society.

Tipping is a significant phenomenon in complex systems^7^, with tipping elements serving as critical components in the Earth system and precursors of global change. These large-scale components may exceed a climate tipping point due to minor perturbations in environmental conditions^8,9^. Once a threshold is crossed, the climate can abruptly shift from one stable state to another, leading to significant and irreversible changes in the Earth system. Such shifts can have dramatic impacts on the biosphere and human societies, including substantial declines in biodiversity, exacerbated global warming, and increased extreme weather events^10^. Understanding the dynamics of tipping elements has garnered considerable attention, as recent research indicates that surpassing 1.5 °C of global warming could activate multiple climate tipping points^11^. Alarmingly, nine tipping points have already been triggered in recent decades, highlighting the urgency of preventing further activation and studying the responses of the Earth system^12^.

The Earth system is significantly influenced by anthropogenic activities, marking the advent of the Anthropocene^13^. Tipping elements of the Earth system will change under the varying climatic conditions triggered by human activities, including the global climate pledges proposed by human society. In 2015, the United Nations Framework Convention on Climate Change (UNFCCC) adopted the Paris Agreement, with 196 parties committing to submit national plans for mitigating climate change. They agreed to regularly review and improve their climate strategies and submit new greenhouse gas (GHG) emission reduction targets as nationally determined contributions (NDCs) every five years, aiming to limit the global average temperature to well below 2 °C above preindustrial levels, while striving to maintain the total increase within 1.5 °C^14,15^. In the 26^th^ United Nations Climate Change Conference of the Parties(COP26) in Glasgow in 2021, 154 parties submitted updated NDCs for reducing GHG emissions by 2030, 76 of which adopted long-term strategies(LTSs)^16^. The Glasgow Climate Pact calls for greater ambition to limit the global average temperature to below 1.5 °C by the end of this century^17,18^.

Global climate pledges significantly influence land use and land cover (LULC). These pledges can mitigate global climate change to some extent while altering various aspects of the Earth, with LULC being a vital and external response. Complex interactions occur between land and climate through biophysical and biogeochemical feedback mechanisms^19–21^. Changes in temperature and precipitation patterns affect land‒atmosphere interactions and biogeochemical cycles, altering the physical properties of land^22–24^ and driving irreversible changes in LULC^25^, land productivity^26^, land suitability^27^, and land-use structures^28^. LULC changes reflect the relationship between climate change and human activities. The ongoing impacts of global warming on LULC have significant direct effects on humans, leading to deforestation, desertification, and the degradation of arable land^29,30^. Additionally, extreme climate events threaten food security, ecological integrity, biodiversity, and human survival^25,31–35^.

Although tipping elements on Earth have been examined from various perspectives, more research is needed on land change projections, particularly in the context of global climate pledges. Studies employing different Earth system models (ESMs) have analysed the temperature thresholds and early warning signals for activating tipping elements^36,37^, as well as the complex interactions and cascading dynamics among these elements^38,39^. They have also investigated the mechanisms and driving factors that lead to tipping points^37,38^, the influence of individual or multiple tipping elements on the global terrestrial ecosystem^40,41^, and the relationship between tipping elements and risk management in socioeconomic development^42,43^. However, the mechanisms of land changes associated with tipping elements under climate change and the potential consequences of future LULC changes have yet to be systematically evaluated. Moreover, numerous global and regional LULC dataset projections exist under different socioeconomic and climate scenarios^44–49^. These scenarios are aligned with the SSP-RCP framework and utilize standardized categories to describe LULC types. It remains to be seen how land types and intensity of terrestrial tipping elements of the Earth system will respond to the proposed global climate pledges.

Determining future land demand is a priority in land change projections. Integrated assessment models (IAMs), which are commonly used to predict global land-use demand under various scenarios, can systematically and quantitatively simulate interactions between Earth systems and human systems, while comprehensively analysing feedback from multiple natural elements on Earth under climate change. Notable IAMs include the Global Change Assessment Model (GCAM)^50^, the Asia–Pacific Integrated Model (AIM)^51^, the Global Biosphere Management Model (GLOBIOM)^52^, and the Model of Agricultural Production and its Impact on the Environment (MAgPIE)^53^. However, significant inconsistencies exist between the results of IAMs and LULC datasets, as the land-use outputs of IAMs often differ in magnitude and type from those of standard LULC datasets. Additionally, land types with similar names may have inconsistent definitions, complicating the correspondence between IAM outputs and LULC data. For example, the GCAM classifies 27 land use categories from an agricultural economics perspective, which does not align with typical LULC datasets^54,55^. Current studies typically rely on manual harmonization of these datasets, using methods such as adjusting the percentage of corresponding land types in LULC datasets and IAMs^45^, analysing the rate of change in IAM results^49^, or applying simple reclassification to establish a mapping relationship^44^. However, these approaches often transform GCAM results to fit LULC datasets, altering quantity and type without directly utilizing the original GCAM predictions, which may introduce uncertainties or errors in projections. To address these challenges, we adopted a proposed concept of a land system^56–59^, which allows for the direct use of GCAM data in land change simulations. A land system is a mixed-type LULC created by reclassifying traditional LULC datasets on the basis of the proportion of land area per unit cell^57^. The land system differs from the present LULC reclassification by more effectively reflecting the type and intensity of land use, thereby better capturing local heterogeneity and specific service functions^59^. The land system comprises a combination of 10 basic land types—cropland, forest, grassland, shrubland, wetland, water body, tundra, artificial surfaces, bare land, and snow or ice—along with three localized density types (low, medium, and high), resulting in a total of 30 distinct types.

**Fig. 1:**
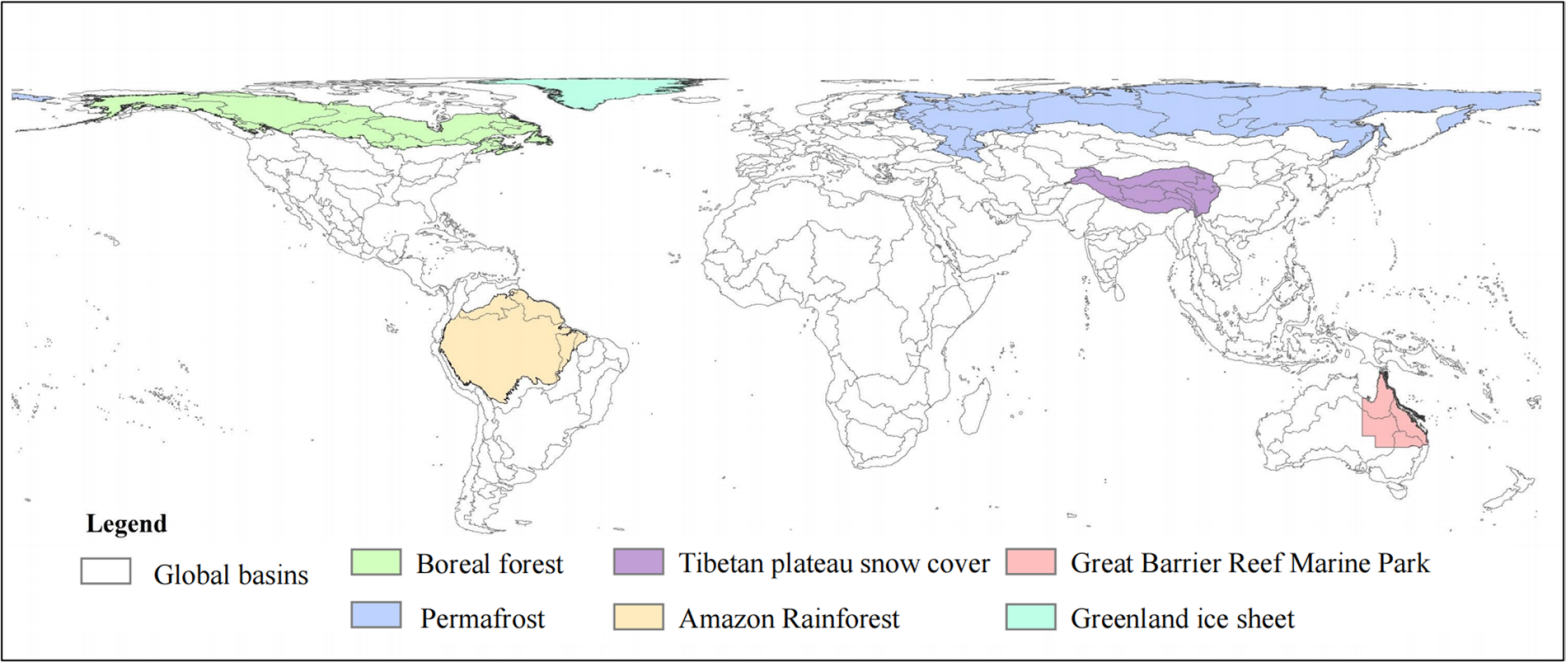
Research area of terrestrial tipping elements on Earth in our study.

We focus on the terrestrial tipping elements on Earth, including the Greenland ice sheet, permafrost, boreal forest, coral reef, Amazon Rainforest, and Tibetan Plateau snow cover. Our research area includes the land surrounding the Great Barrier Reef Marine Park, the largest coral reef in the world^60^, to study changes in land adjacent to shallow coral reefs. The research area is illustrated in Fig. 1, and the land boundary data sources for each tipping element region are detailed in Table 1. Given the geographic heterogeneity of these regions and the divisions in the GCAM, water basins are used as the smallest unit for studying land system changes associated with each tipping element. In total, 64 water basins from the GCAM that overlap with these tipping elements are included.

**Table 1:** Boundary data sources of terrestrial tipping elements on Earth.

Terrestrial tipping elements	Boundary data source
Greenland ice sheet	University of California, Berkeley: Museum of Vertebrate Zoology (https://maps.princeton.edu/catalog/stanford-sd368wz2435)
Permafrost	National Cryosphere Desert Data Center (https://www.ncdc.ac.cn/portal/metadata/e8992bb6-2a89-4be4-b604-457b0164b3f5)
Boreal forest	The official website of the government of Canada (https://natural-resources.canada.ca/our-natural-resources/forests/sustainable-forest-management/boreal-forest/north-american-boreal-zone-map-shapefiles/14252)
Great Barrier Reef Marine Park	Great Barrier Reef (GBR) Features from the eAtlas website (https://eatlas.org.au/data/uuid/ac8e8e4f-fc0e-4a01-9c3d-f27e4a8fac3c)
Amazon Rainforest	The Amazon Biome from the Data Basin website (https://databasin.org/datasets/7c01a6d864fe4158b455c812ab040b1f/)
Tibetan Plateau snow cover	National Tibetan Plateau/ Third Pole Environment Data Center (10.11888/Geogra.tpdc.270099)

We integrated the GCAM with a modified version of the CLUMondo model^57,59^ to create a land system dataset that illustrates the land cover and intensity types under global climate pledges. First, we generated a land system dataset for terrestrial tipping elements on Earth for 2000, 2010, and 2020. Next, we estimated four land system service demands on the basis of the land outputs from GCAM under various global climate pledge scenarios. We then simulated the land system distribution at a spatial resolution of 1 km via the modified CLUMondo model. The simulation results reflect a balance between land system service supply and demand. This study aims to elucidate how land systems will change in terrestrial tipping elements on Earth when global climate pledges are implemented, providing insights that can help society better navigate climate change and mitigate the risk of catastrophic tipping point consequences.

## Methods

### Overall framework

**Fig. 2:**
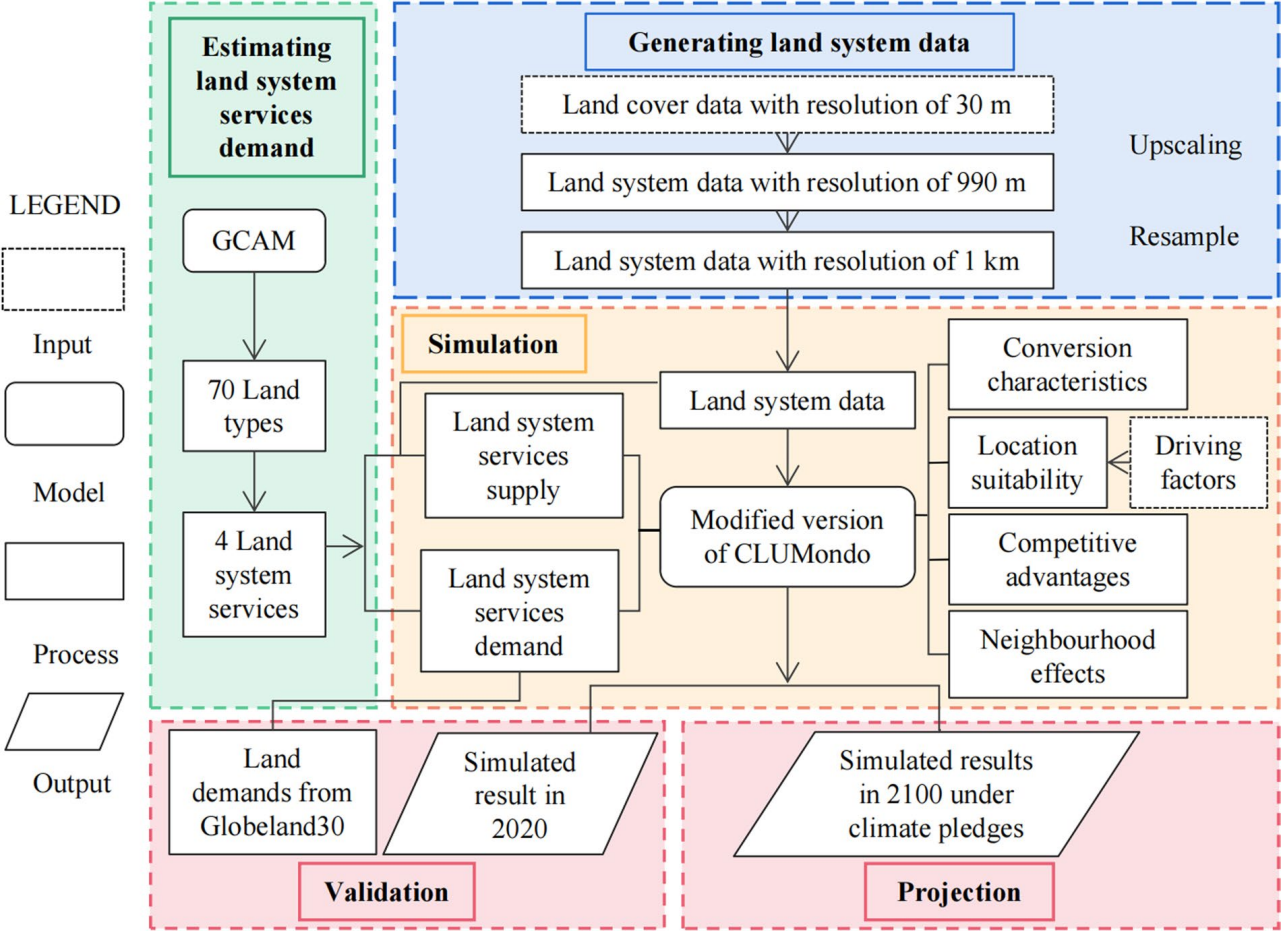
Framework of our study.

The methodological framework for integrating the GCAM and the modified CLUMondo model to produce our dataset is illustrated in Fig. 2. This process can be divided into four parts. The first part involves generating global land system data for the years 2000, 2010, and 2020 from gridded global land cover datasets through upscaling and resampling. The second part focuses on obtaining the demand for land system services. The future demand for land system services is derived from the land output of the GCAM at the water basin level under various global climate pledge scenarios. We reclassify the land types from the GCAM output into four categories of land system services and aggregate the corresponding land areas for these services. The historical demand for land system services, used for accuracy validation, is sourced from global land cover datasets. The third part entails implementing a spatial land system simulation at a resolution of 1 km via a modified version of the CLUMondo model. In this phase, we calculate the location suitability map for all land system types in each grid, determine the supply of land system services for each type, and establish a series of land conversion parameters for the CLUMondo model.

### Generating land system data

The land system maps for 2000, 2010, and 2020 are generated from global land cover data. For this purpose, we selected the GlobeLand30 dataset^61^ (https://www.webmap.cn/commres.do?method=globeIndex), which has a spatial resolution of 30 metres, as the original source. The reasons for this choice are as follows: (1) Spatial resolution: the resolution of this dataset is 30 m, which is superior to that of many existing LULC raster datasets, providing more detailed quantitative and spatial information. (2) Temporal resolution: the dataset encompasses three periods of historical global land cover data—2000, 2010, and 2020—spanning 10-year intervals. This temporal coverage is beneficial for validating land change simulations. (3) Thematic resolution: the dataset includes ten major land cover types (cropland, forest, grassland, shrubland, wetland, water body, tundra, artificial surfaces, bare land, and snow or ice). These classifications encompass the majority of land types used in IAMs, such as the GCAM, ensuring compatibility for our analysis. Some studies similar to our study adopted the European Space Agency Climate Change Initiative (ESA-CCI)^62^ land cover dataset as the simulation base land map^44,46,49,63^. The ESA-CCI provides 300-m resolution annual global surface cover data from 1992 to 2020, and categorizes the surface cover into 22 classes, which can also be considered the input dataset for generating the land system in our study. However, the GlobeLand30 dataset is at 30-m resolution and can provide more detailed information on the spatial distribution of surface cover, which is helpful for capturing the land cover features to aggregate more local cells into a homogeneous unit.

Upscaling is essential for generating land system data from land cover data. Specifically, starting with original land cover data at a spatial resolution of 30 metres (referred to as small cells), we generated larger cell data with a resolution of 990 metres. This was accomplished by applying a sliding window of 33 × 33 cells with a step size of 33 to traverse all small cells. The land types at the 990-m resolution corresponded to the original land cover types with the highest area fractions within the sliding window. For density types, we applied a classification method based on land cover fractions, an approach that has been similarly employed in previous studies to categorize land system types^56,57^,^64–66^. Specifically, we calculated and aggregated the area fractions of the dominant land cover type within larger cells of the same type. Using the natural breaks classification method^67,68^, a widely recognized approach that effectively captures underlying distribution patterns, we identified two breakpoints. This allowed us to categorize the fraction values of each land cover type into three density levels, listed in ascending order: low, medium, and high. The resulting land system data include information on both density and land cover types, such as high-density grassland and medium-density forest. The 990-m resolution data were subsequently resampled to 1 km to create the final land system dataset. Here, we employed the nearest neighbour method, which is the most suitable approach for our study compared with other methods. This method assigns the value of the output raster’s cell to the value of the input raster’s cell that is closest to its center^69^. A key advantage of the nearest neighbour method is that it preserves the original numerical values, making it particularly well-suited for discrete data^70^.

### Land system service demand and supply under global climate pledges

The GCAM is an open-source IAM that predicts future social and environmental scenarios, simulating the interactions between human activities and global change. The core principle of the GCAM is market equilibrium^50^. It encompasses five interconnected systems: energy, water, land, climate, and economy. By inputting historical or future values of key variables across these systems (such as population, income, land, demand and supply for energy, water, food, feed, and forestry), the model can be utilized to obtain results for relevant indicators (such as carbon emissions, water, land, trade, price and quantity of production) under various scenarios from 1990 to 2100 (excluding 2000)^50^. The strengths of the GCAM include the availability of many public and easily accessible GCAM results^71–75^ and its ease of integration with other algorithms or models to simulate future societies and environments from multiple perspectives^76–78^. The GCAM effectively captures the complexities of interactions among these systems and has been widely used in studies of land change simulation in the context of climate change^44,49^. The GCAM categorizes the world into 235 water basins, 384 subregions, and 32 geopolitical regions. The agriculture and land use module within the GCAM reflects the state of agricultural activities and ecosystems, incorporating various land types into its operational results. However, the GCAM has limited spatial visualization capabilities. The quantitative information about land types in subregions lacks detailed spatial information. These numerical results need to be downscaled^79^, thus necessitating the integration of the GCAM with spatial models^44,49,80^.

Future land demand is derived from results obtained in a previous study^71^ via the GCAM. A reference scenario and five emission scenarios—developed under global climate pledges and nationally determined contributions (NDCs)—have been simulated to evaluate the responses of the Earth system, culminating in the calculation of temperature outcome probabilities by 2100. The six scenarios are reference-no policy, current policy, current policy-continued ambition, updated pledges-continued ambition, updated pledges-increased ambition, and the illustrative 50% 1.5 °C scenario. The reference scenario fails to meet the target of limiting temperature rise to 2 °C, whereas the other five scenarios progressively increase the likelihood of achieving either the 2 °C threshold or even the 1.5 °C threshold.

The land type outputs from the GCAM are categorized into four land system services, and the areas of land types are summed to determine the land system service demands, following the approaches of previous studies^57^. The land use output of the GCAM includes 70 land types; however, we excluded the types of tundra, urban, rocky, snow or ice and desert that do not change. The remaining types are reclassified into four common land system services: forestland, grassland/pasture, shrubland, and cropland, as detailed in Table 2. The land system service demand value is the total area of its corresponding land types. The GCAM provides the area of each land type within 235 global water basins. Here, we used the land results from 64 water basins related to terrestrial tipping elements, and calculated the demand of each water basin for the four land system services.

**Table 2:** Categorical relationships between the four land system services and the GCAM land types.

Land system services	Land types in GCAM
Cropland	Corn, Fibre crop, Fodder grass, Fodder herb, Misc crop, Oil crop, Palm fruit, Rice, Root tuber, Sugar crop, Wheat, Biomass grass, Biomass tree, Other grain, Other arable land
Forest	Unmanaged forest, Protected unmanaged Forest, Forest
Grassland	Unmanaged pasture, Protected grassland, Protected unmanaged pasture, Pasture, Grassland
Shrubland	Protected shrubland, Shrubland

**Fig. 3:**
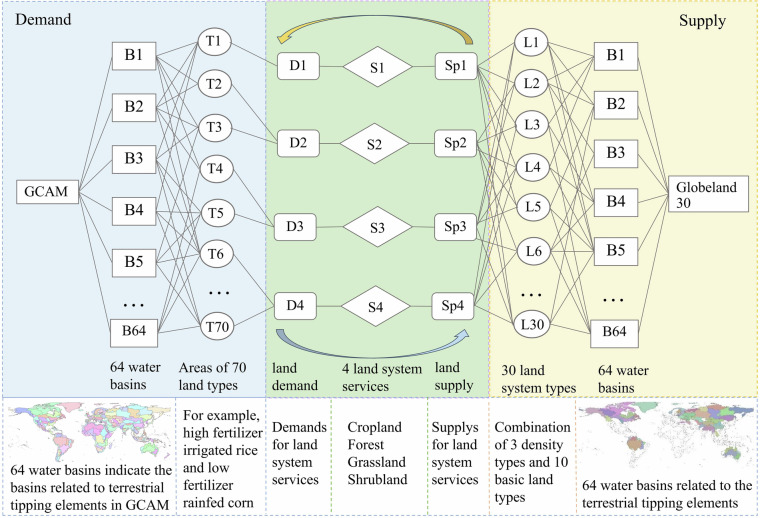
Structure of the many-to-many relationships between land demand and supply through land system services(adapted from ref. ^57^).

We then aggregated the land system data for multiple supply capacity calculations. The four land system services—cropland, forest, grassland, and shrubland—are provided by the small cells at 30-m resolution of the respective land cover types (‘cropland’, ‘forest’, ‘grassland’, and ‘shrubland’). For each land system type, the supply capacity of a unit cell is calculated as the average area of the corresponding 30-m resolution cells within its 990-m resolution cells^81^. This means that the land system service demand comes from the GCAM land areas, whereas the land system service supply capacity is based on the areas of small cells from GlobeLand30. Each of the 30 types of land system can provide a specific area for different land system services (if the value is 0, it is considered not to be provided) under the design of the land system service demand and supply capacity, thereby realizing the many-to-many relationships between land system types and land services (Fig. 3), making the land simulation process and results more scientific.

In this study, we examined two future emission scenarios: the no-policy reference scenario and the updated pledges-continued ambition scenario^71^. The reference scenario, the no-policy scenario, assumes that no new GHG mitigation policies are adopted beyond those already implemented in 2015. In this scenario, near-term trends are driven by energy demand and technology, while the economy primarily influences long-term development^71^. It serves as a benchmark for comparison with other climate pledge scenarios, representing an unlikely outcome with a zero percent chance of limiting the global temperature rise to 2°C or less by 2100. The second scenario is the closest to the implementation of recent global climate pledges, reflecting the impact of countries adopting updated nationally determined contributions and medium- to long-term emission reduction strategies. It is assumed that parties will fulfil their updated climate pledges by 2030. After this point, countries without long-term mitigation strategies maintain the average or lowest carbon reduction rate from 2015 to 2030 (the historical average rate of carbon reduction is less than 2%), whereas those with such strategies follow a defined pathway of emissions reductions leading up to the target year, with subsequent carbon reduction rates on the basis of the historical average between 2015 and the target year^71^. This scenario posits that countries with net-zero emission commitments will achieve their targets by the target year, with emissions remaining constant thereafter, making it the most plausible pathway for achieving near-term goals.

### A modified version of the CLUMondo model

The CLUMondo model is a dynamic land change model that meets exogenous demands through the functional diversity of the land system^59,82^. Its fundamental principle involves modifying land system types through continuous iterations^59^. CLUMondo comprises both external and internal iterations. An external iteration represents the number of simulations conducted from the initial year to the final year and is considered a single external iteration. Each external iteration includes multiple internal iterations, during which one or more cells may change their land system types. The external iteration concludes when the supply of land system services meets the specified demand through internal iterations. While traditional land simulation models construct a single correspondence feature between land area demand and supply^83–85^, the CLUMondo model has a significant advantage in its ability to allow each land system to provide a certain quantity of multiple goods or land services. This establishes many-to-many relationships between land system service supply and demand, enhancing the accuracy and scientific validity of the land change simulation results^86,87^. However, the CLUMondo model still suffers from being unable to simulate successfully because of improper parameter settings in some regions.

CLUMondo consists of both a nonspatial analysis module and a spatial analysis module. The nonspatial analysis module encompasses land use demands and their corresponding supply capacities. We incorporate four types of land system service demands from the GCAM and utilize land system data to calculate supply capacity. The spatial analysis module simulates the land system distribution, with changes influenced by the conversion potential and the following conversion constraints: (1) spatial constraints: certain areas have restrictions that prevent the land system from changing, which are implemented in the model through maps of these constrained areas; (2) type constraints: specific land system types cannot be converted to others. This is represented by a conversion matrix, where 0 indicates that a land system type cannot be converted to another type, and 1 indicates that it can. In this context, we do not consider spatial constraints and assume that between any two different land system types from 2010 to 2020, conversion is represented by 1, whereas the absence of conversion is indicated by 0.

For land cells that are allowed to undergo conversion at a certain time *t*, the change in the land system depends on the conversion potential, prioritizing the cells with the highest potential. The greater the conversion potential is, the higher the priority for conversion. The conversion potential is influenced by factors such as location suitability, competitive advantage, conversion resistance, and neighbourhood effects. The formula is as follows:

$${{\boldsymbol{P\_pot}}}_{{\boldsymbol{c}},{\boldsymbol{j}}}={{\boldsymbol{P\_loc}}}_{{\boldsymbol{c}},{\boldsymbol{j}}}+{{\boldsymbol{P\_res}}}_{{\boldsymbol{T}}({\boldsymbol{c}},{\boldsymbol{t}},0)}+{{\boldsymbol{P\_nei}}}_{{\boldsymbol{c}},{\boldsymbol{j}}}+{{\boldsymbol{P\_comp}}}_{{\boldsymbol{c}},{\boldsymbol{j}}}$$

where $c$ is the *c*-*th* cell and *j* is the *j*-*th* land system type; ${{P\_pot}}_{c,j}$ represents the conversion potential, ${{P\_loc}}_{c,j}$ represents the location suitability, ${{P\_res}}_{T(c,t,0)}$ is the conversion resistance, ${{P\_nei}}_{c,j}$ is the neighbourhood effects, and ${{P\_comp}}_{c,j}$ represents the competitive advantage; *T*(*c*,*t*,0) is the initial land system type of the *c*-*th* cell at moment *t* in the initial land system type.

Location suitability refers to the likelihood that the type of a land system cell will be converted to another type, influenced by various driving factors. A regression analysis of the spatial distribution of the land system using a range of natural, social, and economic drivers is used to estimate the probability that each cell will be converted to a particular land system. The driving factor data we used came from the study^57^, including 65 driving factors in seven categories: soil, socioeconomic, transportation, agriculture and vegetation, climate, topography and density of land cover types. The density of land cover types is calculated on the basis of the area proportion of the corresponding land type in the 1-km land system of each type. Detailed information about the driving factors is listed in Table S1.

Competitive advantage refers to the priority of a land system type to occur conversion, with which a cell can most likely be converted into a type with a higher competitive advantage in comparison with others. Competitive advantage reflects the relative contribution of each land system to certain land system goods or services^59^.

Conversion resistance measures how easily a land system type can be converted to another type, with values ranging from 0 to 1. A higher value indicates greater difficulty in conversion. For example, land types with low reversibility, such as artificial surfaces, exhibit high resistance to conversion. In our study, the conversion resistance of a specific land system is defined as the proportion of that system that does not experience type conversion from 2010 to 2020.

Neighbourhood effects indicate the influence of the portion of land type of surrounding cells on a specific cell. In CLUMondo, neighbourhood weights are assigned to each land system, and the size of the neighbourhood radius can be entered. Through experimental comparison, we found that the validation results without considering neighbourhood effects were better than those with neighbourhood effects (see Text S1 and Table S2 for details). A possible reason for this is that the neighbourhood effect is typically used in simulations of urban land use^88–92^; our study focuses on simulating a comprehensive land system, where urban areas represent only a tiny fraction of the terrestrial tipping elements. Therefore, we did not consider neighbourhood effects, setting their value to 0. We employ a modified version of CLUMondo as proposed in previous studies^57,81^. This version includes three key enhancements over the original model: the introduction of a fine-grained iteration mechanism, an improved regression method for assessing location suitability, and an algorithm for determining competitive advantage^57^.

For the iteration mechanism, the simulation ends if the demand cannot be met after 20,000 iterations in the original CLUMondo and if the simulation does not yield results. The modified model uses a finer-grained iteration method that combines coarse-grained iteration with fine-grained iteration. Specifically, after the initial 20,000 iterations, the model conducts cell-by-cell iterations to facilitate the discovery of a more conclusive simulation result.

Logistic regression was employed to analyse location suitability linearly on the basis of a series of driving factors in the original CLUMondo. However, this statistical method often fails to adequately capture the complex nonlinear relationships between land system patterns and various drivers at larger scales^93^. To address this limitation, the modified CLUMondo introduces a machine learning-based random forest approach, which is more effective at identifying the nonlinear effects of driving factors on land changes and offers enhanced fitting capabilities^83,94^. Therefore, the modified CLUMondo introduced the random forest algorithm to replace the original logistic regression^57^.

Specifically, when calculating the land suitability of the j-*th* type of land system from the starting time *y1* to the ending time *y2*, each sample is represented as a vector comprising a series of driving factors associated with the cells. The driving factors corresponding to the cells that are of type *j* at moment *y1* but not of that type at *y2* are categorized as sample set A, whereas the driving factors for the remaining cells are classified as sample set B. There are 200 decision trees and a 25% sampling proportion. The regression results provide the location suitability of each land cell for the *j*-*th* type, indicating the probability that each cell is suitable for conversion to that type of land system.

The original CLUMondo has a competitive advantage of manually entering the integers, indicating the conversion order for different land system types. A larger value signifies a greater competitive advantage, suggesting that the land system supplies more services and prioritizes conversions during iterations. However, this approach relies heavily on user input, and simple integer values can obscure the nuanced differences in advantages among various land types. To address this issue, the modified CLUMondo introduces an adaptive competitive advantage calculation mechanism. This allows the model to automatically determine the competitive advantage on the basis of the varying capacities of different land types to supply services and the relationship between supply and demand during the simulation, thereby minimizing the need for additional parameter inputs. The specific formulas are as follows:

2 $$\left\{\begin{array}{c}{{\boldsymbol{P\_comp}}}_{{\boldsymbol{c}},{\boldsymbol{j}}}=\sum _{{\boldsymbol{d}}}{{\boldsymbol{inertia}}}_{{\boldsymbol{d}},{\boldsymbol{i}},{\boldsymbol{j}}}\times \frac{{{\boldsymbol{CA}}}_{{\boldsymbol{j}},{\boldsymbol{d}}}-{{\boldsymbol{CA}}}_{{\boldsymbol{u}},{\boldsymbol{d}}}}{{\sum }_{{\boldsymbol{j}}}{{\boldsymbol{CA}}}_{{\boldsymbol{j}},{\boldsymbol{d}}}}\\ {{\boldsymbol{inertia}}}_{{\boldsymbol{d}},{\boldsymbol{i}}}=\left\{\begin{array}{l}{\bf{0}}\,\,\,{\boldsymbol{i}}={\bf{1}}\\ {{\boldsymbol{inertia}}}_{{\boldsymbol{d}},{\boldsymbol{i}}}+\frac{{{\boldsymbol{Demand}}}_{{\boldsymbol{d}}}-{{\boldsymbol{Supply}}}_{{\boldsymbol{d}},{\boldsymbol{i}}-{\bf{1}}}}{{{\boldsymbol{speed}}}_{{\boldsymbol{i}}}}\,{\boldsymbol{i}}\ge {\bf{2}}\end{array}\right.\\ {{\boldsymbol{speed}}}_{{\boldsymbol{i}}}=\left\{\begin{array}{l}{\boldsymbol{seed}}\,{\boldsymbol{i}}={\bf{1}}\\ {{\boldsymbol{speed}}}_{{\boldsymbol{i}}}+{\boldsymbol{step}}\,{\boldsymbol{i}}\ge {\bf{2}}\end{array}\right.\end{array}\right.$$

For the j-*th* and u-*th* land systems, ${{CA}}_{j,d}$ and ${{CA}}_{u,d}$ are the supply capacities of the d-*th* land system service, respectively. ${{Demand}}_{d}$ is the demand for the service of the d-*th* land system, ${{Supply}}_{d,i-1}$ is the supply of the land system at the end of the (i-1)-*th* internal iteration, and ${{inertia}}_{d,i}$ measures the accumulation of the difference between the supply and demand of the service of the d-*th* land system at the end of the (i-1)*th* internal iteration, and is subject to the speed parameter *speed*_*i*_. The ${{speed}}_{i}$ parameter consists of $Seed$ and $St{ep}$, and its value changes as the number of the internal iterations increases. *Seed* is the initial value of *speed*_*i*_. The default value of $Seed$ is 1 and *Step* is 0.001, respectively.

## Data Records

**Fig. 4:**
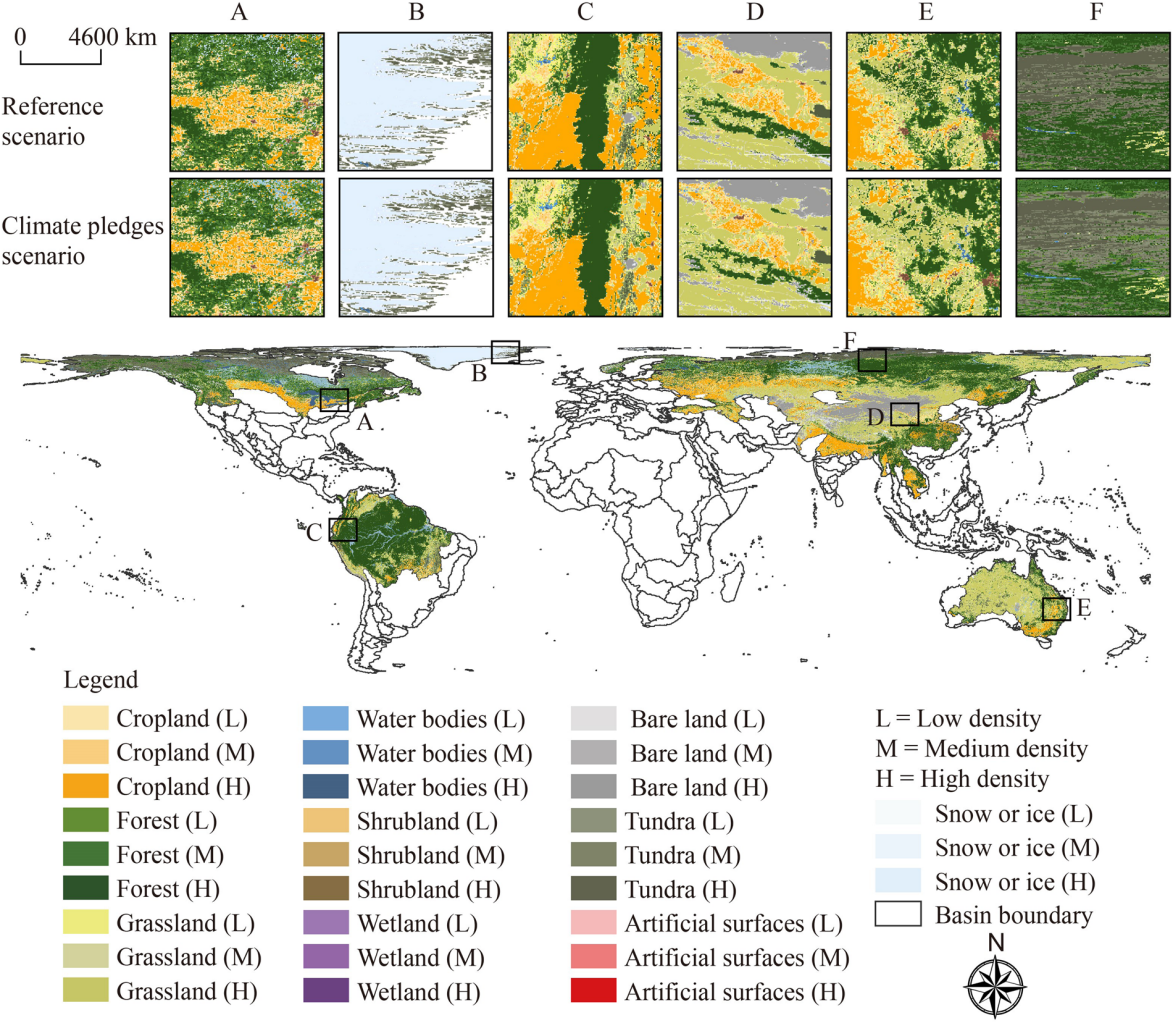
Land system distribution of terrestrial tipping elements in 2100, and a comparison of reference scenarios in selected areas.

Our land system changes dataset can be downloaded from the data repository on FigShare (10.6084/m9.figshare.27087886)^95^. The dataset is provided in a compressed (.zip) file, which includes five maps of a 1-km land system of tipping elements on Earth for the years 2000, 2010, 2020, and 2100 under two scenarios. All files are in GeoTIFF format with a cylindrical equal area spatial reference, allowing easy access through ArcGIS and ENVI, or processing by programming languages such as MATLAB and Python. The naming convention for the files designates the land system data for 2000, 2010, and 2020 as ‘Tipping_elements_YEAR.tif.’ The data for the reference and climate pledges scenarios in 2100 are named ‘Tipping_elements_2100_REF.tif’ and ‘Tipping_elements_2100_PLG.tif’, respectively. The integer values in the raster data represent different land system types, which are detailed in Table 3. We illustrate the distribution of terrestrial tipping elements on Earth for 2100 under the climate pledges scenario and compare it with the reference scenario in selected regions (Fig. 4).

**Table 3:** Land system types corresponding to the integer values in our dataset.

Value	Type	Value	Type
0	Cropland_L	15	Water_L
1	Cropland_M	16	Water_M
2	Cropland_H	17	Water_H
3	Forest_L	18	Tundra_L
4	Forest_M	19	Tundra_M
5	Forest_H	20	Tundra_H
6	Grassland_L	21	Artificial_L
7	Grassland_M	22	Artificial_M
8	Grassland_H	23	Artificial_H
9	Shrubland_L	24	Bare_L
10	Shrubland_M	25	Bare_M
11	Shrubland_H	26	Bare_H
12	Wetland_L	27	Snow/ice_L
13	Wetland_M	28	Snow/ice_M
14	Wetland_H	29	Snow/ice_H

‘L’ denotes the low-density type, ‘M’ denotes the medium-density type, and ‘H’ denotes the high-density type. ‘Water’ denotes water bodies, ‘Artificial’ denotes artificial surfaces, and ‘Bare’ denotes bare land.

## Technical validation

### Accuracy of the land system change simulation

We conducted a historical simulation for technical validation. We simulated the land system changes in 64 water basins related to terrestrial tipping elements on Earth from 2010 to 2020 and calculated accuracy metrics to validate the long-term simulation performance of our methods. Specifically, we used the 2020 GlobeLand30 dataset to derive the total area of four land cover types—cropland, forest, grassland, and shrubland—for the demand for the corresponding land system services. Next, we calculated the supply capacity of each type of land system via the 2010 GlobeLand30 data. We considered that the driving factors do not change over time. By inputting the 2010 land system map, we generated a simulated land system map for 2020. To evaluate the simulation accuracy, we used Kappa coefficient^96^ and the Figure of Merit (FoM)^97^ to compare the simulated map with the land system map derived from the 2020 GlobeLand30 dataset.

The Kappa coefficient measures the similarity between the simulated land system map and the land system data generated from the observed land cover data. A Kappa coefficient closer to 1 indicates higher simulation accuracy. The formula for the Kappa coefficient is presented in Eq. (3):

3 $$\text{Kappa}=\frac{\left({P}_{0}-{P}_{c}\right)}{\left(1-{P}_{c}\right)}$$

where ${P}_{0}$ is the ratio of correctly simulated cells of the totals, namely, the proportion of the overall correct simulation. ${P}_{c}$ is the proportion of correctly simulated cells in the random case.

FoM represents the ratio of correctly predicted changes to the total number of observed and predicted changes, providing an effective measure of the accuracy and correctness of land change simulations^98^. The FoM ranges from 0 to 1, with values closer to 1 indicating better simulation accuracy. The formula for the FoM is presented in Eq. (4).

4 $${\rm{FoM}}=\frac{{\rm{B}}}{{\rm{A}}+{\rm{B}}+{\rm{C}}+{\rm{D}}}$$

A denotes the area that changed in reality but not in the simulation, B denotes the area that changed in reality and in the simulation to the correct type, C denotes the area that changed in reality and in the simulation but to the wrong type, and D denotes the area that did not change in reality but changed in the simulation^99^.

The thematic resolution of the simulation results initially comprised 30 classes. To refine our analysis, we merged classes with the same land type but differing densities, resulting in a thematic resolution of 10 classes. We validated the simulation maps at the basin scale and for both thematic resolutions of 30 and 10 classes. Table 4 presents the Kappa coefficients and FoM for the historical land simulations. The average Kappa coefficient for 30 classes is 83.29%, with an FoM of 7.46%. When the thematic resolution is reduced to 10 classes, the average Kappa coefficient increases to 90.82%, and the FoM increases to 15.55%.

**Table 4:** Accuracy of historical simulations for each water basin related to terrestrial tipping elements on Earth. ‘_30’ indicates validation at 30 thematic resolutions, whereas ‘_10’ refers to validation at 10 thematic resolutions.

Basin name	Kappa_30	FoM_30	Kappa_10	FoM_10
Arctic Ocean Islands	79.76%	7.07%	90.25%	11.66%
Northwest Territories	79.10%	27.52%	89.54%	27.52%
Siberia North Coast	89.45%	7.45%	97.39%	18.44%
Siberia West Coast	87.09%	2.42%	94.08%	8.46%
Kara Sea Coast	87.43%	6.66%	97.33%	11.29%
Lena	88.01%	12.90%	95.29%	16.68%
Pacific and Arctic Coast	84.07%	6.30%	92.59%	17.90%
Scandinavia North Coast	86.06%	11.05%	94.69%	17.74%
Russia Barents Sea Coast	84.82%	7.90%	93.56%	25.41%
Mackenzie	84.04%	9.65%	92.32%	20.78%
Finland	83.91%	3.51%	91.63%	3.50%
Northern Dvina	76.76%	1.43%	84.57%	7.68%
Hudson Bay Coast	84.55%	13.74%	92.86%	31.56%
Neva	86.22%	6.32%	94.00%	11.74%
Volga	83.51%	0.83%	92.44%	3.25%
Atlantic Ocean Seaboard	84.66%	17.03%	91.71%	28.75%
Baltic Sea Coast	86.64%	2.43%	93.98%	5.44%
Narva	86.98%	6.94%	93.68%	15.41%
Saskatchewan Nelson	85.85%	1.00%	93.62%	3.41%
Daugava	83.86%	5.54%	92.39%	13.62%
Fraser	68.94%	0.17%	71.72%	3.63%
Poland Coast	84.36%	3.85%	92.08%	13.88%
Churchill	86.42%	6.43%	92.19%	7.37%
Neman	83.10%	4.35%	91.43%	13.90%
Russia South East Coast	86.96%	3.01%	92.46%	6.43%
Ural	89.54%	6.93%	94.88%	13.40%
Dnieper	83.08%	0.57%	91.36%	2.42%
St Lawrence	85.63%	3.16%	91.84%	7.68%
Gobi Interior	85.25%	14.29%	91.73%	24.92%
Amur	86.96%	5.70%	93.98%	12.35%
Caspian Sea Coast	89.30%	8.87%	94.33%	17.03%
Black Sea North Coast	55.49%	1.38%	54.87%	2.07%
Yenisey	88.45%	1.58%	95.01%	5.14%
Don	68.19%	17.87%	85.34%	16.49%
Ob	86.72%	1.73%	94.54%	4.37%
Amu Darya	91.48%	9.69%	96.42%	25.78%
Black Sea South Coast	51.92%	5.47%	76.42%	6.67%
Caspian Sea South West Coast	86.18%	1.97%	93.28%	6.24%
Syr Darya	89.53%	2.01%	94.21%	7.20%
Huang He	81.81%	4.56%	90.98%	13.96%
Tarim Interior	76.32%	5.35%	84.56%	8.30%
Indus	87.52%	10.35%	94.12%	15.74%
Plateau of Tibet Interior	79.71%	7.14%	87.47%	17.77%
Yangtze	84.25%	6.38%	92.69%	21.35%
Ganges Brahmaputra	80.71%	9.83%	90.26%	25.23%
Mekong	83.44%	0.46%	87.83%	1.32%
Salween	82.92%	23.36%	91.10%	49.05%
Irrawaddy	88.90%	3.32%	94.60%	7.40%
Orinoco	85.60%	9.01%	91.61%	18.44%
Colombia Ecuador Pacific Coast	86.57%	16.14%	92.48%	33.77%
Northeast South America South Atlantic Coast	87.57%	5.59%	93.57%	20.12%
Magdalena	79.76%	13.13%	88.11%	33.26%
Amazon	85.49%	8.42%	92.87%	23.34%
North Brazil South Atlantic Coast	72.56%	4.36%	82.37%	14.79%
Tocantins	74.92%	3.16%	84.86%	11.04%
Peru Pacific Coast	89.77%	5.88%	95.46%	15.54%
Australia North Coast	89.22%	17.16%	94.73%	36.92%
Australia East Coast	86.14%	16.92%	91.92%	31.65%
Australia Interior	89.02%	25.09%	93.49%	44.97%
Murray Darling	81.78%	21.46%	89.68%	37.97%
Upper Mississippi Basin	73.29%	1.07%	80.72%	7.20%
Great Lakes Basin	90.29%	0.47%	96.68%	2.57%
Pacific Northwest Basin	84.49%	1.70%	91.42%	5.32%
New England Basin	88.38%	0.10%	92.94%	0.94%
Average	83.29%	7.46%	90.82%	15.55%

Our accuracy validation results outperform those of many similar studies, and the thematic and spatial resolutions of our data are also higher than those of many other studies. This indicates that our data accuracy is credible and that our method performs well in simulating land system changes.

First, our Kappa value is higher than those reported in many publications. In the study by Liu *et al*.^84^ on a LULC simulation in China, the average Kappa coefficient according to the validation was 79.63%. Cao *et al*.^80^ reported an average Kappa of 49%. Chen *et al*.^46^ achieved an average Kappa of 86.4%. In the study by Dong *et al*.^54^, the average Kappa used for validation was 79%.

Moreover, our FoM is higher than that in many other studies. The average FoM in the research by Liu *et al*.^84^ was 12.46%. Luo *et al*.^49^ simulated future LULC changes in China, and the verified average FoM was 13%. Zhang *et al*.^44^ obtained a verified average FoM of 10% for land changes in China. Chen *et al*.^46^ reported an average FoM of 10.2%. The average FoM in the study^54^ was 12.14%.

In addition, our thematic and spatial resolutions are also higher than those in many other studies. For example, the spatial resolution was 1 km, and the thematic resolution was six classes in the study^84^. Cao *et al*.^80^ produced a land dataset with a spatial resolution of 1 km and a thematic resolution of ten classes. Luo *et al*.^49^ simulated land changes with a spatial resolution of 1 km and a thematic resolution of eight classes. Zhang *et al*.^44^ generated land change data for China with a 1 km resolution and six LULC types. Chen *et al*.^46^ produced a 1-km land cover dataset that classified seven land types; its spatial resolution was 5 km, and its thematic resolution was five classes^100^. Dong *et al*.^54^ published a 1-km dataset with six land classes.

## Usage Notes

This study presents a 1-km resolution land system dataset for terrestrial tipping elements on Earth for 2000, 2010, 2020, and 2100 under global climate pledge scenarios. The dataset encompasses 30 land system types, which are derived from a combination of ten basic land types and three density categories. To validate the dataset, we performed a historical simulation at the basin scale, and the results demonstrate its high accuracy.

The dataset is highly applicable to a range of specific research questions and policy-relevant issues. First, it provides a more comprehensive understanding of land changes in terrestrial tipping elements as the Earth approaches climate tipping points. Integrating this dataset with established risk identification methods^43,101^ and various geoanalysis models^102^ can make it a vital tool for policy-makers in the formulation of land use planning and management strategies aimed at monitoring, preventing, and managing the governance of terrestrial tipping elements^103,104^.

Second, the dataset can be integrated with ESMs to model future Earth system dynamics and improve the understanding of the interactions between global climate pledges and the Earth system under climate change. For example, previous studies^45,100^ have classified future land use under SSP and RCP scenarios into plant functional types that are consistent with the Community Land Model version 5 (CLM5)^105^. The global future land use/cover dataset has also been integrated with the common land model (CoLM)^106^ to predict global gross primary productivity^107^.

Furthermore, our dataset can be combined with biodiversity datasets^108^, species distribution models^109^ and landscape ecological analyses^110,111^ to predict species distributions, identify priority areas for biological conservation, and support ecological conservation and habitat restoration efforts under global climate pledge scenarios.

## Supplementary information

Supplementary information

## Data Availability

The source code for the modified version of CLUMondo used in this paper is available at Zenodo (10.5281/zenodo.7661313)^112^. The dataset has also been uploaded to figshare. Any additional information needed to reanalyse the data reported in this paper can be obtained from the corresponding author upon request.
