# Publisher Correction: Land system changes of terrestrial tipping elements on Earth under global climate pledges: 2000–2100

**DOI:** 10.1038/s41597-025-04648-y

**Published:** 2025-02-20

**Authors:** Jiaying Lv, Yifan Gao, Changqing Song, Li Chen, Sijing Ye, Peichao Gao

**Affiliations:** State Key Laboratory of Earth Surface Processes and Resource Ecology, Beijing Normal University, Beijing, 100875 China

**Keywords:** Environmental impact, Projection and prediction, Environmental impact, Projection and prediction

Correction to: *Scientific Data* 10.1038/s41597-025-04444-8, published online 27 January 2025

The Acknowledgments section was missing from this article and should have read ‘This study was supported by the National Natural Science Foundation of China (Grant Nos. 42230106, 42271418, 42171250, and 42171088)’. The original article has been corrected.

