# Supplementary material for: Land system changes of terrestrial tipping elements on Earth under global climate pledges: 2000–2100
Source: Sci Data. 2025 Jan 27;12:163. doi: 10.1038/s41597-025-04444-8 (PMC11772770; doi:10.1038/s41597-025-04444-8)
Supplement: Supplementary file 1 — Supplementary information [file 41597_2025_4444_MOESM1_ESM.pdf]

Supplementary Information of

Land system changes of terrestrial tipping elements on Earth under global climate pledges: 2000-2100

Authors
Jianyig Lv[1], Yifan Gao[1], Changqing Song[1], Li Chen[1], Sijing Ye[1], Peichao Gao[1]

Affiliations
1. State Key Laboratory of Earth Surface Processes and Resource Ecology, Beijing Normal University, Beijing, 100875, China.
corresponding author: Peichao Gao (gaopc@bnu.edu.cn)

Table of Contents

Texts

Text S1 Supplementary explanation for the experiment where neighbourhood information was not considered in the simulation

In this study, we did not consider neighbourhood effects. The incorporation of neighbourhood information is indeed a common practice in land system modelling. The modified CLUMondo model also supports this feature; we are aware of it and have made efforts to apply it in our simulations. However, after careful consideration of the neighbourhood effects, we found that including this factor actually led to less accurate simulation results.

We conducted several simulation experiments for a single basin, both with and without the inclusion of neighbourhood effects, and compared the resulting accuracy metrics. In CLUMondo, it is possible to define neighbourhood weights and radii for each land type. We used a set of common parameter settings for incorporating neighbourhood information, where all land system types were assigned a neighbourhood weight of 1, with neighbourhood radii set to 1, 2, and 3.

Our comparison of the results (Table S2) revealed that the best simulation outcomes, in terms of Kappa and FoM, were achieved when the neighbourhood effect was not considered. Specifically, when the land system had 30 categories and no neighbourhood effect was applied, the Kappa value was 89.54% and the FoM was 6.93%. In contrast, for the experimental groups where the neighbourhood effect was considered, the Kappa value ranged from 89.14% to 89.23%, and FoM ranged from 2.22% to 3.21%. When the land system was simplified to 10 categories, the Kappa and FoM values without considering the neighbourhood effect were 94.88% and 13.40%, respectively. For the groups with neighbourhood effects, the Kappa coefficient ranged from 94.42% to 94.55%, and the FoM ranged from 5.64% to 7.34%.

The primary reason for our differing approach is that we simulate a comprehensive full-range land system changes, whereas the neighbourhood effect is typically applied in simulations of urban land use, such as urban expansion[1-3]. In the case of the terrestrial tipping elements we focus on, urban areas are relatively sparse, which makes neighbourhood effects unsuitable for our simulation.

Tables

Table S1. The driving factor information we used was from a previous[4]

Driving factor	Resolution	Year	Data source
Bulk density	7.5 arc-sec	2017	Hengl *et al*.[5]
Cation exchange capacity	7.5 arc-sec	2017	
Clay content	7.5 arc-sec	2017	
Coarse fragments volumetric	7.5 arc-sec	2017	
Derived available soil water capacity	7.5 arc-sec	2017	
Organic carbon density	7.5 arc-sec	2017	
pH in H2O	7.5 arc-sec	2017	
Sand content	7.5 arc-sec	2017	
Silt content	7.5 arc-sec	2017	
Texture class	7.5 arc-sec	2017	
Market access index	5 arc-min	2011	Verburgt *et al*.[6]
Market influence index ($/person)	5 arc-min	2011	
Market density index	5 arc-min	2011	
Nighttime lights	30 arc-sec	2010	Version 4 DMSP-OLS Nighttime Lights Time Series (https://www.ngdc.noaa.gov/eog/dmsp/downloadV4composites.html)
Total GDP (PPP, purchasing power parity)	30 arc-sec	2015	Kummu *et al*.[7]
Gridded Population of the World (GPW)	30 arc-sec	2010	Gridded Population of the World, Version 4 (GPWv4): Population Density, Revision 11 (https://earthdata.nasa.gov/data/catalog/sedac-ciesin-sedac-gpwv4-popdens-r11-4.11#ds-description-collapse)[8]
Time to nearest cities	30 arc-sec	2015	Weiss *et al*.[9]
Distance to nearest river	1 km	N/A	Calculated based on the rivers_lake_centerlines vectors from Natural Earth (https://www.naturalearthdata.com/downloads/10m-physical-vectors/10m-rivers-lake-centerlines/)
Distance to nearest road	1 km	N/A	Calculated based on the road vectors from Natural Earth (https://www.naturalearthdata.com/downloads/10m-cultural-vectors/roads/)
Distance to nearest railway	1 km	N/A	Calculated based on the railroad vectors from Natural Earth (https://www.naturalearthdata.com/downloads/10m-cultural-vectors/railroads/)
Travel time one metre (motorized)	30 arc-sec	2019	Weiss *et al*.[10]
Travel time one metre (walking-only)	30 arc-sec	2019	
Time to nearest health care facility (motorized)	30 arc-sec	2019	
Time to nearest health care facility (walking-only)	30 arc-sec	2019	
175 Crops Yield per hectare	5 arc-min	2000	Monfreda *et al*.[11]
Gross primary production (March)	3 arc-min	2010	Wang *et al*.[12]
Gross primary	3 arc-min	2010	

production (June)			
Gross primary production (September)	3 arc-min	2010	
Gross primary production (December)	3 arc-min	2010	
Normalized Difference Vegetation Index (March)	~1 km	2010	MYD13A2 MODIS/Aqua Vegetation Indices 16-Day L3 Global 1km SIN Grid[13]
Normalized Difference Vegetation Index (June)	~1 km	2010	
Normalized Difference Vegetation Index (September)	~1 km	2010	
Normalized Difference Vegetation Index (December)	~1 km	2010	
Elevation	1 arc-sec	N/A	Fick, S. E. & Hijmans, R. J.[14]
Variance of elevation	1 km	N/A	Calculated based on elevations
Slope	1 km	N/A	
Aspect	1 km	N/A	
Annual mean precipitation	30 arc-sec	2007-2018avg	Monthly precipitation in mm at 1 km resolution based on SM2RAIN-ASCAT 2007-2018, IMERGE, CHELSA Climate and WorldClim (0.2)[15]
Monthly mean precipitation (March)	30 arc-sec	2007-2018avg	
Monthly mean precipitation (June)	30 arc-sec	2007-2018avg	
Monthly mean precipitation (September)	30 arc-sec	2007-2018avg	
Monthly mean precipitation (December)	30 arc-sec	2007-2018avg	
Annual mean temperature	30 arc-sec	2000-2017avg	Long-term MODIS LST day-time and night-time temperatures, sd and differences at 1 km based on the 2000–2020 time series (1.0)[16]
Monthly mean temperature (March)	30 arc-sec	2000-2017avg	
Monthly mean temperature (June)	30 arc-sec	2000-2017avg	
Monthly mean temperature (September)	30 arc-sec	2000-2017avg	
Monthly mean temperature (December)	30 arc-sec	2000-2017avg	
Buffaloes	5 arc-min	2010	Gilbert *et al.*[17]
Cattle	5 arc-min	2010	Gilbert *et al.* [18]
Chickens	5 arc-min	2010	Gilbert *et al.*[19]
Ducks	5 arc-min	2010	Gilbert *et al.*[20]
Goats	5 arc-min	2010	Gilbert *et al.*[21]

Horses	5 arc-min	2010	Gilbert *et al*.[22]
Pigs	5 arc-min	2010	Gilbert *et al*.[23]
Sheep	5 arc-min	2010	Gilbert *et al*.[24]
Fraction of ‘cultivated land’ micro cells	1 km	2010	Calculated based on the 2010 land cover dataset of GlobeLand30
Fraction of ‘forest’ micro cells	1 km	2010	
Fraction of ‘grassland’ micro cells	1 km	2010	
Fraction of ‘shrubland’ micro cells	1 km	2010	
Fraction of ‘wetland’ micro cells	1 km	2010	
Fraction of ‘water bodies’ micro cells	1 km	2010	
Fraction of ‘tundra’ micro cells	1 km	2010	
Fraction of ‘artificial surfaces’ micro cells	1 km	2010	
Fraction of ‘bareland’ micro cells	1 km	2010	
Fraction of ‘snow/ice’ micro cells	1 km	2010	

Table S2. Validation metrics for simulations with and without considering neighbourhood effects.

<table>
<tr><th colspan="2">Neighbourhood effects settings</th><th>Kappa_30</th><th>FoM_30</th><th>Kappa_10</th><th>FoM_10</th></tr>
<tr><td colspan="2">No neighbourhood effects</td><td>89.54%</td><td>6.93%</td><td>94.88%</td><td>13.40%</td></tr>
<tr><td rowspan="3">Neighbourhood weight value = 1</td><td>Neighbourhood radius = 1</td><td>89.16%</td><td>2.22%</td><td>94.46%</td><td>5.64%</td></tr>
<tr><td>Neighbourhood radius = 2</td><td>89.14%</td><td>2.73%</td><td>94.42%</td><td>6.50%</td></tr>
<tr><td>Neighbourhood radius = 3</td><td>89.23%</td><td>3.21%</td><td>94.55%</td><td>7.34%</td></tr>
</table>

References

1 Hagoort, M., Geertman, S. & Ottens, H. Spatial externalities, neighbourhood rules and CA land-use modelling. *The Annals of Regional Science* **42**, 39-56, doi: 10.1007/s00168-007-0140-8 (2008).

2 van Vliet, J. *et al.* Measuring the neighbourhood effect to calibrate land use models. *Comput. Environ. Urban Syst.* **41**, 55-64, doi: https://doi.org/10.1016/j.compenvurbsys.2013.03.006 (2013).

3 Zhao, Y., Cui, B. & Murayama, Y. Characteristics of neighborhood interaction in urban land-use changes: A comparative study between three metropolitan areas of Japan. *J. Geogr. Sci* **21**, 65-78, doi: 10.1007/s11442-011-0829-6 (2011).

4 Gao, P. *et al.* Fulfilling global climate pledges can lead to major increase in forest land on Tibetan Plateau. *iScience* **26**, 106364, doi: https://doi.org/10.1016/j.isci.2023.106364 (2023).

5 Hengl, T. *et al.* SoilGrids250m: Global gridded soil information based on machine learning. *PLoS One* **12**, (2017).

6 Verburg, P. H., Ellis, E. C. & Letourneau, A. A global assessment of market accessibility and market influence for global environmental change studies. *Environ. Res. Lett.* **6**, 034019, doi: 10.1088/1748-9326/6/3/034019 (2011).

7 Kummu, M., Taka, M. & Guillaume, J. H. A. Gridded global datasets for Gross Domestic Product and Human Development Index over 1990–2015. *Sci. Data* **5**, 180004, doi: 10.1038/sdata.2018.4 (2018).

8 Gridded Population of the World, Version 4 (GPWv4): Population Density, Revision 11. *Socioeconomic Data and Applications Center (SEDAC)* https://doi.org/10.7927/h49c6vhw (2018).

9 Weiss, D. J. *et al.* A global map of travel time to cities to assess inequalities in accessibility in 2015. *Nature* **553**, 333-336, doi: 10.1038/nature25181 (2018).

10 Weiss, D. J. *et al.* Global maps of travel time to healthcare facilities. *Nat. Med.* **26**, 1835-1838, doi: 10.1038/s41591-020-1059-1 (2020).

11 Monfreda, C., Ramankutty, N. & Foley, J. Farming the Planet: 2. Geographic Distribution of Crop Areas, Yields, Physiological Types, and Net Primary Production in the Year 2000. *Global Biogeochem. Cycles* **22**, GB1022, doi: 10.1029/2007GB002947 (2008).

12 Wang, S., Zhang, Y., Ju, W., Qiu, B. & Zhang, Z. Tracking the seasonal and inter-annual variations of global gross primary production during last four decades using satellite near-infrared reflectance data. *Sci. Total Environ.* **755**, 142569, doi: https://doi.org/10.1016/j.scitotenv.2020.142569 (2021).

13 Didan, K. MYD13A2 MODIS/Aqua Vegetation Indices 16-Day L3 Global 1km SIN Grid. *NASA Land Processes Distributed Active Archive Center(LP DAAC)* http://doi.org/10.5067/MODIS/MYD13A2.006 (2015).

14 Fick, S. E. & Hijmans, R. J. WorldClim 2: new 1‐km spatial resolution climate surfaces for global land areas. *Int. J. Climatol.* **37**, 4302-4315, doi: https://doi.org/10.1002/joc.5086 (2017).

15 Hengl, T. Monthly precipitation in mm at 1 km resolution based on SM2RAIN-ASCAT 2007-2018, IMERGE, CHELSA Climate and WorldClim (v0.2). *Zenodo* https://doi.org/10.5281/zenodo.3256275 (2018).

16 Hengl, T. Long-term MODIS LST day-time and night-time temperatures, sd and differences at 1 km based on the 2000–2017 time series (v1.0). *Zenodo* https://doi.org/10.5281/zenodo.1435938 (2018).

17 Gilbert, M. *et al.* Global buffaloes distribution in 2010 (5 minutes of arc) (v3). *Harvard Dataverse* https://doi.org/10.7910/DVN/5U8MWI (2018).

18 Gilbert, M. *et al.* Global cattle distribution in 2010 (5 minutes of arc) (v3). *Harvard Dataverse* http://dx.doi.org/10.7910/DVN/GIVQ75 (2018).

19 Gilbert, M. *et al.* Global chickens distribution in 2010 (5 minutes of arc) (v3). *Harvard Dataverse* https://doi.org/10.7910/DVN/SUFASB (2018).

20 Gilbert, M. *et al.* Global ducks distribution in 2010 (5 minutes of arc) (v3). *Harvard Dataverse* https://doi.org/10.7910/DVN/ICHCBH (2018).

21 Gilbert, M. *et al.* Global goats distribution in 2010 (5 minutes of arc) (v3). *Harvard Dataverse* https://doi.org/10.7910/DVN/OCPH42 (2018).

22 Gilbert, M. *et al.* Global horses distribution in 2010 (5 minutes of arc) (v3). *Harvard Dataverse* https://doi.org/10.7910/DVN/7Q52MV (2018).

23 Gilbert, M. *et al.* Global pigs distribution in 2010 (5 minutes of arc) (v3). *Harvard Dataverse* https://doi.org/10.7910/DVN/33N0JG (2018).

24 Gilbert, M. *et al.* Global sheep distribution in 2010 (5 minutes of arc) (v3). *Harvard Dataverse* https://doi.org/10.7910/DVN/BLWPZN (2018).
